# The role of subjective significance, valence and arousal in the explicit processing of emotion-laden words

**DOI:** 10.7717/peerj.14583

**Published:** 2023-01-06

**Authors:** Kamil K. Imbir, Joanna Duda-Goławska, Adrianna Wielgopolan, Adam Sobieszek, Maciej Pastwa, Jaroslaw Zygierewicz

**Affiliations:** University of Warsaw, Warszawa, Polska, Polska

**Keywords:** Emotion-laden words, Emotional categorisation, Explicit word meaning processing, P300, LPC, N400

## Abstract

Emotional categorisation (deciding whether a word is emotional or not) is a task that employs the explicit analysis of the emotional meaning of words. Therefore, it allows for assessing the role of emotional factors, *i.e*., valence, arousal, and subjective significance, in emotional word processing. The aim of the current experiment was to investigate the role of subjective significance, a reflective form of activation that is similar to arousal (the automatic form), in the processing of emotional meaning. We applied the orthogonal manipulation of three emotional factors. Thus, we were able to precisely differentiate the effects of each factor and search for interactions between them. We expected valence to shape the late positive complex LPC component, while subjective significance and arousal were expected to shape the P300 and N400 components. We observed the effects of subjective significance throughout the whole span of processing, while the arousal effect was present only in the LPC component. We also observed that amplitudes for N400 and LPC discriminated negative from positive valence. The results showed that all factors included in the analysis should be taken into account while explaining the processing of emotion-laden words; especially interesting is the subjective significance, which was shown to shape processing individually, as well as to come into interaction with valence and arousal.

## Introduction

### Emotional factors in word processing

The emotional categorisation task is intuitive for individuals taking part in experiments. It provides a unique opportunity to search for the impact caused by emotional factors such as valence, arousal, and subjective significance on the explicit processing of meaning, giving us a chance to understand the affect structure. The most important description of emotional states is valence (Russell, 1980, 2003; Barrett, 1998), which indicates pleasantness or non-pleasantness of events or certain stimuli. Positive valence is a general term describing pleasurable and satisfying emotional states, such as joy or relaxation, while negative valence, in general, describes the wide range of feelings usually avoided by people, such as sadness or anger (Osgood, Suci & Tannenbaum, 1957; Kagan, 2010). Positively valenced stimuli have been shown to promote processing in generalised, global terms, faster reactions and decisions, and less accurate responses (Fredrickson, 1998; Fredrickson & Branigan, 2005; Imbir et al., 2020a). On the other hand, negatively charged stimuli promote focusing on details, but may also increase reaction times compared to neutral stimuli (Hinojosa, Méndez-Bértolo & Pozo, 2010; Palazova, Sommer & Schacht, 2013; Gupta, Hur & Lavie, 2016; Imbir et al., 2020a, 2021a). Both positive and negative valence might also be applied to concepts (objects) and constitute an affect assigned to them, being an appraisal on how pleasant, neutral or unpleasant a given stimulus is Russell (1980); a similar appraisal procedure is used during the creation of affective norms for words (*e.g*., Bradley & Lang, 1999).

Emotional arousal is the dimension describing the activation and energy towards some object (Russell, 1980, 2003; Russell & Barrett, 1999); it can be evoked by both positive and negative stimuli, as it is the term describing general emotional charge brought by a certain feeling (Russell, 2003). It is also rather automatic, very much connected with physiological functioning and innate reactions (Imbir et al., 2017). Highly pleasurable and unpleasurable stimuli can be described as arousing, but this description can also fit sexual stimuli or stimuli simply related to movement or action, not easily appraised in terms of valence (Ariely & Loewenstein, 2006). For example, hearing an unexpected loud noise might be activating, but it is hard to quickly assess whether the noise itself is good or bad. The arousal evoked in the subject is simply a reaction of the autonomic nervous system, which is not necessarily tied to particular emotions. According to Yerkes–Dodson law, optimal level of arousal should improve processing, while too high or too low arousal may impair it (Yerkes & Dodson, 1908; Teigen, 1994). This seems to be in line with recent experiments exploring the influence of arousal evoked by words on performance in cognitive tasks—highly arousing stimuli tend to slow down reactions and decisions as well as reduce the accuracy in cognitive tasks (Yao et al., 2016; Imbir et al., 2020a).

The last interesting factor—very recently proposed (Imbir, 2015, 2016a)—is the subjective significance (van Hooff et al., 2008; Imbir et al., 2017). The subjective significance is a dimension of an emotional activation, being the dual-process theories’ answer to the question about activation not being the monolithic dimension (Thayer, 1986; Watson et al., 1999), but rather a construct which might be further separated into specific types of activation. Furthermore, it was a construct present in the narrative about emotions for some time—for example, Averill 1975; already wrote about the property of emotions called the ‘depth of experience’, this being the dimension on how profound, significant and important was a feeling (as opposed to being shallow and insignificant). Separating the arousal and subjective significance accordingly to the dual-process theories might explain the difference between them; in a way, the subjective significance may be perceived as a reflective version of arousal; however, it is worth noting that those two dimensions are rather independent (weakly correlated with each other (Imbir, 2016b)), and should be treated as such. In contrast to automatic arousal, subjective significance requires effortful and complex cognitive processing of stimuli. It might be described as an attitude towards the object in relation to the situation, goals and values of an individual; it is an assessment of whether there is a need to invest in systematic, reflective processing (Imbir, 2016a, 2016b). As a result, the subjective significance load of an object gives an individual a sense of what is important. Certain objects evoking emotions may have a specific load of significance (especially for a particular population, the same as for arousal). Stimuli with a high level of subjective significance tend to be related to ideas perceived as important to society, such as family or religion, and worth engaging in Koole & Coenen (2007) and Imbir (2016a). Therefore, subjective significance might be a reliable measure to describe stimuli which are not easy to assess as equivocally positive or negative, but also are not as basic and biological as the high arousing ones, requiring rather cognitive processing, for example, getting a well-paid job might be perceived as a neutral (as a thing one has to do) but highly subjective significant (in order to achieve some other goals). Studies exploring the subjective significance factor have reported its influence on the accuracy and speed of processing in cognitive tasks—with the increase of subjective significance level, the decrease in reaction times and increase in accuracy were observed (Imbir et al., 2020a). It also shapes both implicit and explicit processing of emotion-laden words’ meaning—in studies including EEG measurement, highly significant words resulted in less negative amplitudes in the FN400 component (Imbir et al., 2018a, 2020b). Lastly, a valid concern is whether subjective significance of stimuli can be reliably measured, and whether it is separate from other previously proposed emotional factors, such as valence, arousal and dominance. Imbir (2016c) found ratings of subjective significance to be both reliable and exhibiting a weak correlation with the aforementioned emotional dimensions (0.16, 0.38, and 0.23, respectively, while the correlation between valence and arousal was found to be 0.46).

### Emotional word processing stages

From an event-related potential (ERP) perspective, emotional word processing can be studied using two different basic approaches (Citron, 2012; González-Villar et al., 2014). First are studies employing involuntary word processing tasks, such as the emotional Stroop task, where participants are asked to name the colour of the font an emotional word is written in Pérez-Edgar & Fox (2003), Thomas, Johnstone & Gonsalvez (2007) and Imbir et al. (2017). Although the execution of the task does not require reading the word, this process still occurs involuntarily. There, we see modulation occurring mostly in early components, starting 200 ms after the stimuli onset, such as the early posterior negativity effect (Herbert et al., 2006; Kissler et al., 2007; Schacht & Sommer, 2009). Second are studies that require explicit, more in-depth processing of words. They reveal a different activation pattern that is seen mostly in later components associated with semantic analysis of the word’s meaning (Palazova, Sommer & Schacht, 2013) and attention to task demands (Fischler & Bradley, 2006; Hajcak, MacNamara & Olvet, 2010). Moreover, among these types of tasks, we can differentiate between subtypes according to whether the task requires explicit processing of the word’s emotive content. The first subtype comprises tasks that require explicit processing of the word, but do not draw the participants’ attention to its emotionality (*e.g*., the lexical decision task; Imbir et al., 2020b), whereas the second subtype is made up of tasks involving assessment of the word’s emotive content. The emotional categorisation task situates itself in the latter category, as it requires participants to perform deeper, semantic processing of words and explicit assessment of the emotional load of words. Thus, we shall consider in detail the components of interest in such a case: the P300, N400, and the late positive complex (LPC).

The P300, a component present in the time window between 250 to 350 ms, is a positive deflection occurring in the centro-parietal regions of the scalp (Picton, 1992). It is usually studied in oddball tasks, in which participants watch target and standard stimuli; in this paradigm, P300 is significantly more positive for target stimuli than for standard ones. This effect seems to be related to the process of categorising the stimuli and reacting to the arousal caused by the new ones (Hajcak, MacNamara & Olvet, 2010). P300 is affected by the probability of stimuli appearance and their importance, *i.e*. more positive amplitudes were observed in this component for more meaningful stimuli (Johnson, 2007; Thomas, Johnstone & Gonsalvez, 2007). More positive amplitudes were also reported in the P300 time window for emotional items (rather than non-emotional), which could be explained by more effortful and complex processing of this kind of stimuli (Hajcak, MacNamara & Olvet, 2010). Finally, P300 is also susceptible to the valence of the stimulus (Conroy & Polich, 2007; Scott et al., 2009). This may be observed especially in the case of negative (threatening) emotional words, for which the amplitude of P300 was more positive in comparison to neutral words. However, this effect was significantly smaller when word meaning (no matter the valence) was not relevant to the task (Thomas, Johnstone & Gonsalvez, 2007).

The N400 is a negative deflection, the peak of which could be observed around 400 ms stimulus onset around centro-parietal areas of the scalp (Kutas & Dale, 2013; Bridger et al., 2012), however its localisation and exact peak could vary in different studies (Kutas & Federmeier, 2011). It was originally observed occurring in response to linguistic stimuli, when one part of the stimulus (*e.g*., sentence, in which the second part is semantically incorrect) does not match the other (Kutas & Hillyard, 1980), and has been since frequently observed in response to semantic properties of linguistic stimuli (Lau, Phillips & Poeppel, 2008; Kutas & Federmeier, 2011). The component could be understood as the indicator of novelty, confusion or incongruence (Kutas & Federmeier, 2000, 2011). The N400 component could be observed in experiments requiring decisions, as identifying the semantic novelty or confusing value of a stimulus is an important factor in the decision-making process (Debruille, Pineda & Renault, 1996; Yang & Zhang, 2011; Bridger et al., 2012; Hundrieser & Stahl, 2016; Ozkara & Bagozzi, 2021). Studies have shown that emotional properties of stimuli can influence the N400 potential, with emotional words evoking more negative N400 amplitudes than the neutral ones in tasks requiring cognitive processing (Kanske & Kotz, 2007; Gootjes et al., 2011; Kanske, Plitschka & Kotz, 2011; Blomberg et al., 2020), as well as decision-making tasks (Yang & Zhang, 2011). This difference could be attributed to the arousing value of emotions, not depending on the valence charge, which could be supported by studies showing the influence of arousal on this component (Zhang, Kong & Jiang, 2012). An interesting study has shown that plain emotionality of words may reduce the influence of context congruence on N400, regardless of the valence of the emotional words (Delaney-Busch & Kuperberg, 2013). When it comes to specific effects of valence on N400, negative emotions tend to evoke more negative deflections than positive ones, as negative concepts may seem more unfamiliar to participants than positive ones (Federmeier et al., 2001; Kanske & Kotz, 2007; De Pascalis et al., 2009; Yao et al., 2016). In previous studies conducted by our team employing a procedure requiring emotional decisions we observed effects of emotional factors in N400-like components. Highly arousing stimuli evoked more negative waves than medium and low arousing ones (Imbir et al., 2018b), also negative stimuli evoked more negative amplitudes than positive ones (Imbir et al., 2019), which seems to be in line with other described findings. It is important to note that we also observed the effects of subjective significance in the N400-like component, with highly significant words evoking more positive waves than the medium or low ones on the subjective significance scale (Imbir et al., 2018b).

The last component of interest is the late positive complex (LPC). It is located in the parietal area, and its amplitude peaks between 500 and 800 ms (Citron, 2012). Early studies of emotional word processing reported inconsistent, even contradictory results regarding LPC effects (Cuthbert et al., 2000; Herbert et al., 2006; Herbert, Junghofer & Kissler, 2008; Kanske & Kotz, 2007; Kissler et al., 2007; Schacht & Sommer, 2009), but as the LPC is said to be a manifestation of later stages of semantic processing (Sass et al., 2010; Zhang et al., 2014), it is associated with a conscious recognition of and attention to the stimulus (Hajcak, MacNamara & Olvet, 2010). Additionally, one regularity seems to find more robust support in the literature: the LPC becomes more emotionally modulated as the level of attention to the word’s emotionality increases (Hinojosa, Méndez-Bértolo & Pozo, 2010; González-Villar et al., 2014). For example, González-Villar et al. (2014) conducted a study using the emotional Stroop task and the emotional decision task. They found that the LPC was modulated by valence and arousal only in the latter of the tasks. Nevertheless, many studies have found valence effects within the LPC component (Herbert et al., 2006; Kanske & Kotz, 2007; Herbert, Junghofer & Kissler, 2008; Hofmann et al., 2009; Kissler et al., 2009; Schacht & Sommer, 2009; Gootjes et al., 2011). We can also expect emotional arousal to influence this component (Delaney-Busch, Wilkie & Kuperberg, 2016). Some reports also suggest that self-relevance of the stimuli can influence this stage of processing (Herbert et al., 2011; Herbert, Pauli & Herbert, 2011), which in the context of our study could support predictions regarding the effects of subjective significance.

Novelty brought by the study proposed in this article lies in manipulating simultaneously all three aforementioned emotional factors, namely subjective significance, valence and arousal, while controlling for length of words and frequency of use. This approach allows not only the exploration of potential interactions between the factors, but also to check whether results regarding particular factors of emotional processing are independent of the influence of other factors. In most of our previous experiments exploring emotional words’ properties we used 2-factorial design (*e.g*., Imbir et al., 2017, 2018b), and the 3-factorial design recently incorporated by our research team has already been proven reliable in cognitive tasks (Imbir et al., 2021a, 2021b). What is more, this study is one of the first to explore how subjective significance affects ERPs during decision-making, with a previous study reporting only exploratory results of subjective significance producing ERP modulation during emotional categorisation (Imbir et al., 2018b).

### Aim and hypothesis

In the current experiment, we investigated the role of emotional factors such as subjective significance, valence and arousal in the processing of emotional words during explicit emotional categorisation. We applied unique orthogonal manipulations of three emotional factors to investigate the role of individual factors and interactions between factors. From those three factors it was especially interesting to us to investigate the effects of subjective significance in different ERP components—ERP measurements give us an opportunity to verify the influence of different factors on processing with high accuracy regarding the timeline of components. This allows us to verify whether processing the properties of stimuli regarding significance take place before, during or after processing of emotional valence and arousal.

At the electrophysiological level, we expected to find effects of emotional factors for components associated with word meaning processing, namely P300, N400, and LPC. For the P300 component, we expected to find amplitude to be more positive in high-intensity conditions in contrast to low-intensity conditions for factors of subjective significance and arousal. In the case of the N400 component, associated with the detection of unexpected stimuli (Kutas & Federmeier, 2011), we predicted a dissociation of arousal and subjective significance effects. Namely (1) a more negative amplitude for weakly subjectively significant stimuli in comparison to highly subjectively significant words, and (2) a more negative amplitude for highly arousing stimuli when compared to poorly arousing words (Kanske & Kotz, 2007; Gootjes et al., 2011; Kanske, Plitschka & Kotz, 2011; Blomberg et al., 2020). At the LPC component, we expected to find differences in amplitudes between negative and positive words since, in the literature, the valence levels are differentiated at this component (Citron, 2012); we were also predicting replication of the pattern of subjective significance and arousal from the P300 component, namely increasingly positive amplitudes with the increase of the levels of those emotional factors (Herbert et al., 2011; Herbert, Pauli & Herbert, 2011). At the behavioural level, we expected that valenced (negatively and positively), highly arousing and highly subjectively significant words would be interpreted as more emotional in comparison to neutrally valenced words low in arousal and subjective significance.

## Materials and Methods

### Participants

To determine the number of participants needed for reliable results, we conducted *a priori* analyses using G-Power software (Faul et al., 2007). We used the *η*_*p*_^*2*^ values from previous experiments using emotional words and the same task as in the present experiment (Imbir et al., 2018b, 2019). In earlier studies we obtained *η*_*p*_^*2*^ for ERPs ranging from 0.06 to 0.20 for single emotional factor and from 0.05 to 0.12 for the interaction of two emotional factors. Using these values we estimated that to achieve the effect size of *η*_*p*_^*2*^ = 0.10 for the interaction of two emotional factors with a high statistical power of 0.80, we would need at least 36 participants. However, in the present experiment, we wanted to explore the influence of three emotional factors simultaneously and verify potential interactions among all those factors, which is why we decided to extend the sample size to 48 participants.

The participants were recruited from various faculties of Warsaw universities. They received a small payment for taking part in the experiment. The inclusion criteria were to be right-handed, a native Polish speaker, without chronic clinical issues that may affect EEG recording directly or through medication, intact vision, or corrected to normal by glasses. The entire experimental group consisted of 48 subjects (23 men and 25 women), from 18 to 30 years old (*M* = 22.69; *SD* = 3.21). After pre-processing of the data, two subjects had to be excluded from further analysis. The exclusion criteria arising from the data quality are described below (see *Offline EEG signal processing*) Effectively, there were 46 participants included in the further analysis, 23 men and 23 women, aged 18–29 years (*M* = 22.48; *SD* = 3.07).

Data were collected as previously described in Imbir et al. (2021a). We did not collect any personal data that would allow the identification of the participants. The participants provided informed consent to participate in the experiment, and the fact was documented in a research diary (Imbir et al., 2021a). The bioethical committee of the Faculty of Psychology at the University of Warsaw approved the design, experimental conditions, and procedure. All of the procedures involving human participants were conducted following the institutional and national research committee’s ethical standards (Imbir et al., 2021a) and with the 1964 Helsinki Declaration and its later amendments or comparable ethical standards.

### Design

We manipulated three factors: subjective significance (three levels), valence (three levels) and arousal (three levels) while controlling the following properties of words: frequency of appearance in language and length. We used an orthogonal design, in which each of the manipulated factors is crossed, such that effects of one factor can be assessed independent of the others. This allows us to estimate each main effect, as well as all possible two-way and three-way interactions. Such a design, however, requires a careful selection of stimuli, such that there are no interactions between the ratings of the manipulated factors in all combination of factor levels. This is discussed in the following section, and shown rigorously in Appendix 1.

### Linguistic materials

We obtained emotional words from the Affective Norms for Polish Words Reloaded database (Imbir, 2016c), which is a database of 4,900 Polish words accompanied by eight different affective measures, including arousal, valence, and subjective significance. The database was established in a study, where for each factor 50 participants, half of whom were women, were asked to assess all words on one dimension using the Self-Assessment Manikin (SAM) scale; each scale ranging from one (*e.g*., *negative, non-arousing*) to nine (*e.g*., *positive, strongly arousing*). These ratings were then converted into means for every scale.

The design of the present study involved 27 conditions (3 × 3 × 3 design); for each condition we selected words, which differed in levels of arousal (low, moderate, or high), valence (negative, neutral, or positive) and subjective significance (low, moderate, or high), but not in any of the controlled factors, namely word length (number of letters) and frequency of use (Kazojć, 2011). We selected 15 nouns for each condition for a total of 405 words. The descriptive statistics of manipulated and controlled variables in each manipulation are summarized in Table 1. Examples of words used in each of the groups are presented in Table 2.

**Table 1:** Descriptive statistics of the manipulated and controlled variables for each level of valence, arousal and subjective significance manipulation.

		Valence		Arousal		Subjective Significance		Length		Frequency	
		*M*	*SD*	*M*	*SD*	*M*	*SD*	*M*	*SD*	*M*	*SD*
Valence	Negative	3.96	0.60	4.08	0.63	3.65	0.61	6.99	2.14	5.42	1.72
	Neutral	5.11	0.31	4.02	0.65	3.63	0.64	6.63	2.05	5.66	1.73
	Positive	6.13	0.47	4.00	0.71	3.70	0.65	6.70	2.15	5.57	1.72
Arousal	Low	5.10	0.98	3.37	0.38	3.66	0.64	6.73	2.19	5.60	1.70
	Medium	5.01	1.02	3.96	0.21	3.65	0.66	6.81	2.11	5.64	1.86
	High	5.09	1.02	4.76	0.42	3.68	0.60	6.78	2.05	5.41	1.60
Subjective significance	Low	5.05	0.94	4.00	0.64	3.02	0.31	6.47	2.05	5.30	1.74
	Medium	5.10	0.99	4.08	0.70	3.62	0.18	6.78	2.09	5.55	1.75
	High	5.05	1.09	4.02	0.67	4.35	0.43	7.07	2.18	5.80	1.66

**Table 2:** Word examples for each of the 27 experimental groups, both in English and Polish language (in brackets). The emotional charge of exact words may differ in Polish and English language, the exact values for each word on emotional scales are available in the Appendix 1.

Subjective significance	Valence	Arousal		
		Low	Medium	High
Low	Negative	*Gap* *(Luka)*	*Smoke* *(Dym)*	*Gamble* *(Hazard)*
	Neutral	*Wire* *(Drut)*	*Agency* *(Agencja)*	*Judo* *(Dżudo)*
	Positive	*Breeze* *(Bryza)*	*Lottery* *(Loteria)*	*Gallop* *(Galop)*
Medium	Negative	*Routine* *(Rutyna)*	*Nonsense* *(Nonsens)*	*Witch* *(Czarownica)*
	Neutral	*Level* *(Poziom)*	*Fashion* *(Moda)*	*Fetish* *(Fetysz)*
	Positive	*Autumn* *(Jesień)*	*Cartoon* *(Kreskówka)*	*Comedy* *(Komedia)*
High	Negative	*Apathy* *(Apatia)*	*Control* *(Kontrola)*	*Insult* *(Zniewaga)*
	Neutral	*Distance* *(Dystans)*	*Habit* *(Nawyk)*	*Budget* *(Budżet)*
	Positive	*Wheather* *(Pogoda)*	*Writer* *(Pisarz)*	*Virgin* *(Dziewica)*

Appendix 1 details the ANOVA analyses performed, which show the validity of the stimuli. In short, the analyses showed that when we divide the stimuli into groups based on one of the manipulated factors, there is a significant effect only of the corresponding emotional dimension (*e.g*., groups divided by valence differ only in valence ratings). The analyses also showed no significant interaction effects, as none of the three possible two-way interactions between factors (valence and significance, arousal and significance, valence and arousal) was statistically significant, either for emotional or control dimensions. Finally, we did not find a three-way interaction between arousal, valence and significance on any of the dimensions. All 405 words used in this study along with their affective measures can be found in Appendix 1.

### Procedure

The subjects sat in a comfortable chair, while the words were displayed on a 17.3-inch LCD, positioned approximately 1 m from the subject’s eyes. The font was Helvetica, with a size of 10 per cent of the screen height. Participants were encouraged to respond as quickly as possible.

The subject’s task was to judge whether the presented word was emotional or non-emotional. Before the task they were presented with some brief information paragraph, which introduced them to the concept of a word being ‘emotional’ or ‘non-emotional’. The paragraph of text described how emotional words could be positive, negative, how the arousal they cause may be independent from other aspects, and how other words are non-emotional. It was followed by a graph presenting the span of emotionality, with more emotional concepts (negative, positive, but also independently arousing) far from the neutral (non-emotional) concepts. Then, the participants completed a short practice trial of the task (using only neutral words), after which they began the main task; both practice task and the experimental task used the same procedure.

The participants saw one word on a screen and two possible answers (emotional and non—emotional); to answer they were supposed to press one of the two tagged keys on the keyboard, representing assessing the word as emotional or non-emotional. Both the decision and reaction times were recorded. An experimental set of words comprised 405 words and was presented twice in order to obtain 30 trials for each emotional condition (15 words presented twice), as this number would be appropriate for analysing the ERPs (Luck, 2014). Stimuli were displayed in random order. Trials proceeded as follows:

1. fixation cross displayed for a randomly varied interval of between 600 and 700 ms;
2. stimulus displayed until the participant responded, however not shorter than for 300 ms;
3. the blank screen for a randomly varied interval of between 700 and 800 ms.

**Figure 1:**
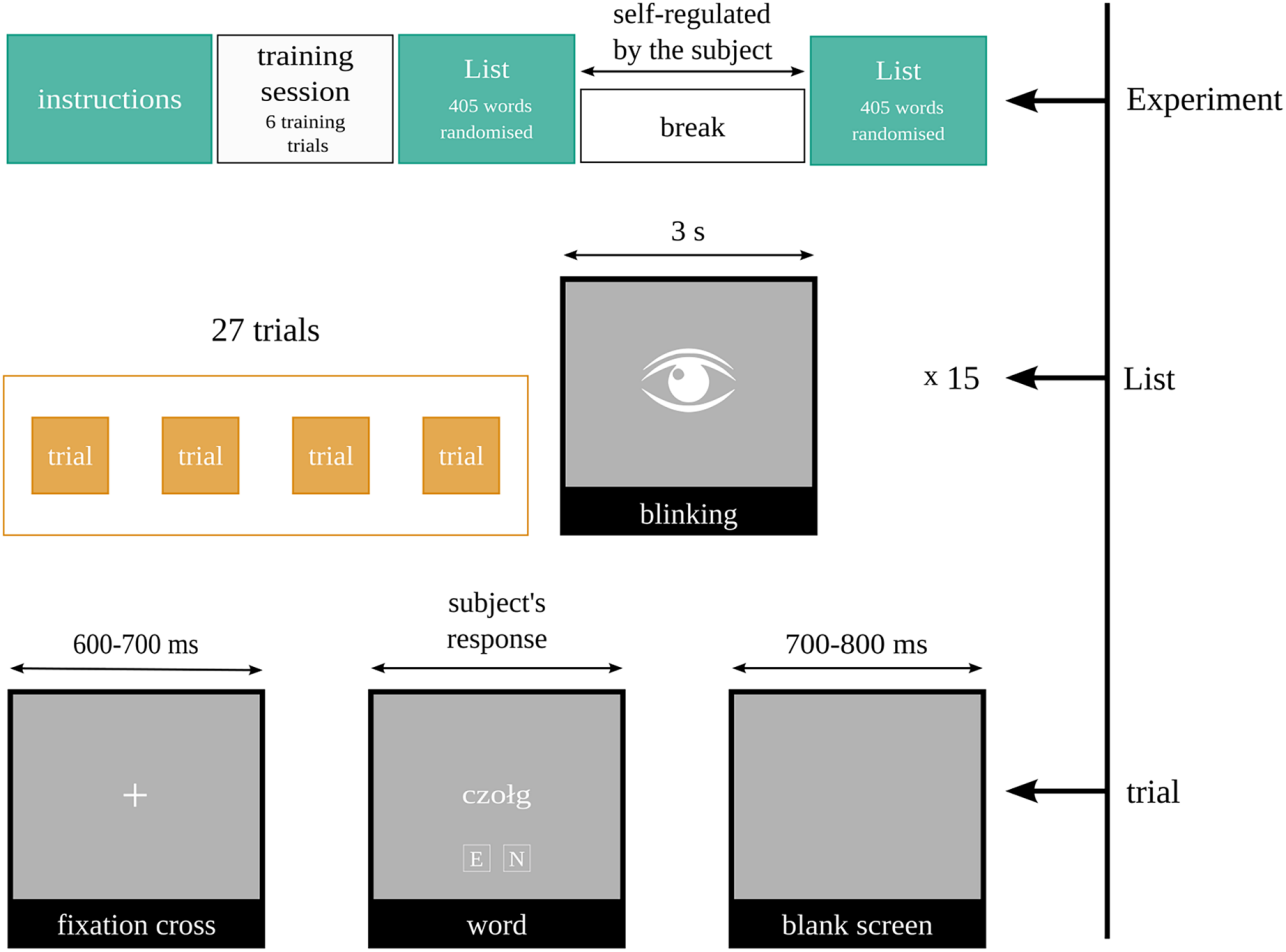
Diagram of the experimental protocol. The task was to assess if the word was emotional or neutral.

The experimental protocol provided three-second breaks for blinking every 27 trials. The procedure is sketched in Fig. 1.

### EEG recording

#### Apparatus

The stimuli were displayed on a standard personal computer monitor. The stimuli were synchronised to EEG data utilising a circuit that recorded the brightness of a small rectangle on display, covered from the subject’s view. Its brightness changed synchronously with the content of the screen. We recorded EEG signals from 19 electrode sites: Fz, Cz, Pz, Fp1/2, F7/8, F3/4, T7/8, C3/4, P7/P8, P3/4, O1/2 referenced to linked earlobes, grounded at the AFz position. All impedances were kept at a similar value below 5 kOhm. The signal was acquired using a Porti7 (TMSI) amplifier, sampled at 1,024 Hz.

#### Offline EEG signal processing

We conducted the offline signal processing utilising Matlab® with the EEGLAB toolbox (Delorme & Makeig, 2004) and custom-made scripts. Offline, the signal was zero-phase filtered, the high-pass cut-off 0.1 Hz, the low-pass cut-off 30 Hz, the notch for the 49.5–50.5 Hz band, all implemented as second-order Butterworth filters with 12 dB/octave roll-off. We extracted intervals ranging from −200 to 800 ms, with 0 being the onset of the stimulus. Next, the signals were baseline-corrected using −200 to 0 ms interval.

From the further analysis we removed trials with extremely short or long response times (RTs). In the first step, we excluded trials with RTs shorter than 300 ms, as we assume that the faster RTs are due to accidentally pressing the key. The remaining RTs have a skewed distribution; therefore, to analyse them using parametric tests, we performed logarithm transformation. The transformed RTs have an approximately normal distribution. As outliers, we define extreme data, *i.e*., outside the limits of $\rm [Q1-1.5*IQR, Q3+1.5*{\rm IQR}]$ (Q1, Q3 are first and third quartile, IQR = Q3 – Q1). For data from a normal distribution, the probability of lying beyond such range is approximately 0.7%. This procedure was done for each subject separately, as each had its individual distribution of reaction times. Finally, RTs for the analysed data across all the subjects were within the range of 300–9,605 ms.

Trials with an excessive linear trend (threshold slope value exceeding 30 μV/s) or excessive amplitude (outside of the range −65 to 65 μV) were marked as corrupted by artifacts and removed from the analysis. Subjects were excluded from further analysis if they had more than 50% of trials marked as corrupted (two subjects were excluded for this reason). In the analysed data, the mean number of trials per condition was *M* = 29.00, *SEM* = 0.04 for analyses of behavioural data and *M* = 25.46, *SEM* = 0.09 for EEG data.

As mentioned before, in the present study we analysed three components: P300, N400 and LPC. For the P300 components we analysed the signal from Cz and Pz electrodes (as we expected to observe the component in the centro-parietal areas) in the time window from 275 to 325 ms stimulus onset (Picton, 1992). For the N400 component we analysed the signal from Cz and Pz electrodes (as, again, this component was expected to be observed in centro-parietal areas) in the time window from 330 to 470 ms stimulus onset (Kutas & Federmeier, 2011). For the LPC component we analysed the signal from P3, Pz and P4 electrodes (we expected to observe this component in parietal areas) in the time window from 470 to 700 ms stimulus onset (Citron, 2012). For each component we analysed the signal averaged among the electrodes chosen for the particular component. The time windows used in the study were chosen to roughly agree with the values suggested in the literature, but were slightly adjusted based on the grand mean waveform to suit the current data. This, to some extent a data-driven approach, may increase the risk of false positives due to implicit visual comparisons (see Luck & Gaspelin, 2017).

### Statistical procedures

We calculated block-to-block consistency from the answers given by participants. We found that the average proportion of assessing the word in the same manner by one participant in the first and the second block was 0.78, with SD = 0.08, which means that the consistency of assessments was much higher than we would expect for random answers. In the main analyses we investigated the logarithms of reaction times, frequency of emotional decisions (percentage of ‘emotional’ response in each condition), and the EEG components’ amplitudes (the average amplitude within the characteristic time window and region of interest) using ANOVA with repeated measures in a hierarchical procedure. The significant main effects were analysed with paired *t*-tests with Holm’s correction for multiple comparisons (Holm, 1979). The significant two-way interactions were investigated further, using a series of one-way ANOVA with repeated measures within individual levels of the interacting factors, followed by *post hoc* paired *t*-tests with Holm’s correction. The significance of the effects repeatedly appearing in the given series of ANOVAs was corrected for multiple comparisons by the Bonferroni correction. In the case of significant three-way interactions, they were further analysed by a series of two-way ANOVAs with a selected variable set iteratively to each of its levels. The selected variables were permuted. Obtained significant two-way interactions were further investigated using *post hoc t*-tests with Holm’s correction. In case an effect could be obtained by different paths in the hierarchical analysis, we report the most conservative result. The three-way interactions are not described in the main body of the manuscript due to their complexity, but for the sake of completeness of presentation are reported in the Appendix 2.

We checked the sphericity with Mauchly’s test and applied the Greenhouse–Geisser correction where necessary. The procedures were implemented in the R statistical package (R Core Team, 2017).

## Results

### Electrophysiological data

We present the results obtained for classical ERP components known from the literature to be related to emotional decision tasks. All amplitudes *M* and *SEM* are given in μV.

**Figure 2:**
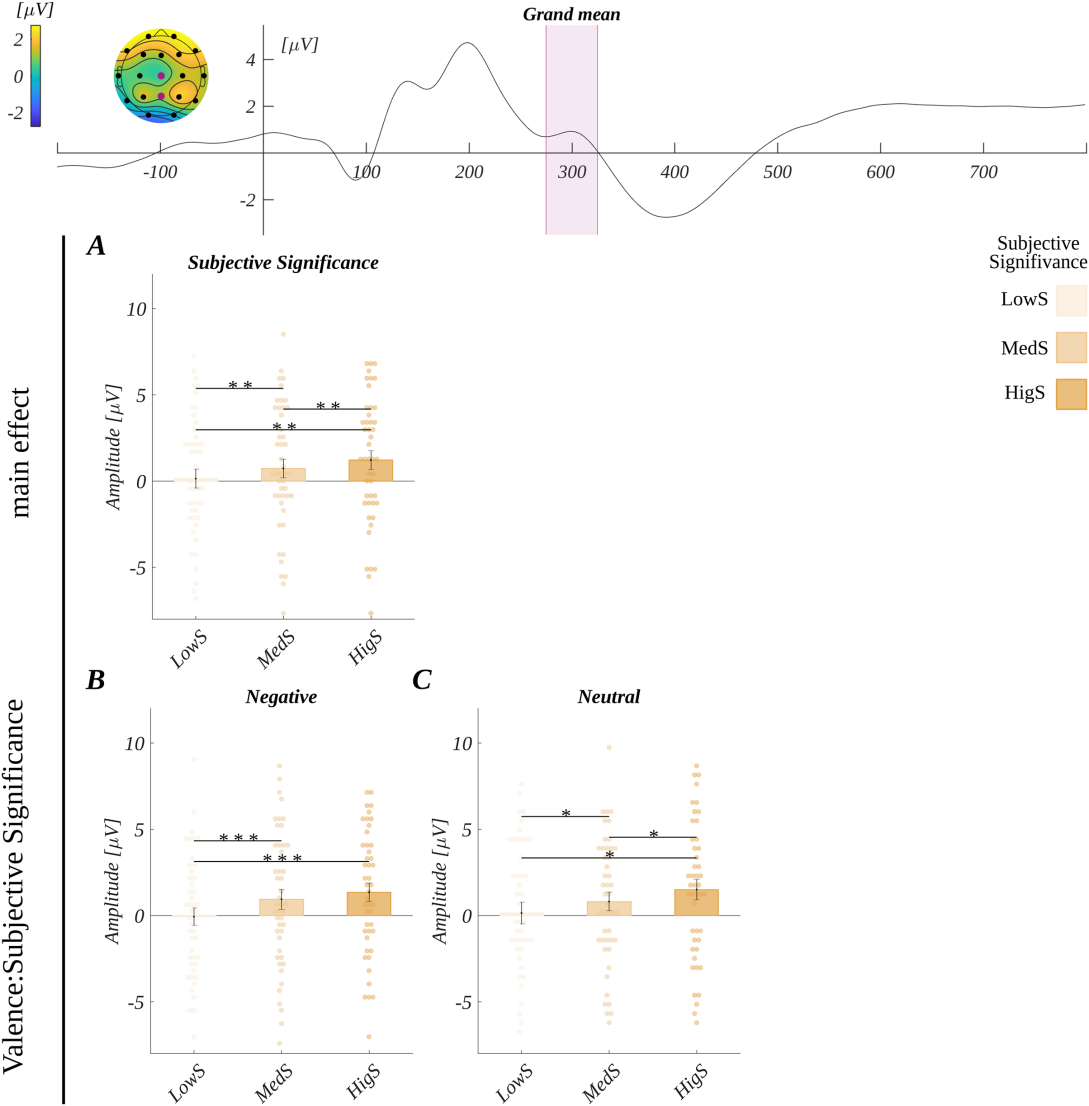
Grand average across electrodes from ROI_P300_ and results for P300 analysis. The upper plot: the grand average across electrodes from ROI_P300_. The marked time-interval was selected for the P300 analysis. The insert shows the topography of the mean potential from this period with the electrodes forming the ROI_P300_ marked with pink dots. Below: the results for P300 analysis. (A) The differences in the amplitude according to the levels of subjective significance. Interaction effects: differences between subjective significance levels for (B) negative, and (C) neutral words. Bars represent the mean value, error bars *SEM*, individual dots mark the amplitude for an individual subject in the given condition, black horizontal lines indicate pairs of conditions with significantly different means (*p < 0.05, **p < 0.01, ***p < 0.001).

The grand average signal from ROI_P300_ is shown in Fig. 2. We obtained the main effect of subjective significance (*F*(2, 90) = 31.15, *p* < 0.001; *η*_*p*_^*2*^ = 0.41). *Post hoc* tests showed that the amplitude increased with the increase of the subjective significance level (Fig. 2A). Specifically, the amplitude for medium significant words (*M* = 0.73, *SEM* = 0.55) was more positive than for the low significant words (*M* = 0.14, *SEM* = 0.55; *t*(45) = 4.26, *p* < 0.001, *d* = 1.27). Furthermore, it was more positive for highly significant words (*M* = 1.22, *SEM* = 0.54) than for medium significant stimuli (*t*(45) = 3.23, *p* = 0.002, *d* = 0.96). Also, the amplitude for highly significant words was more positive than for low significant words (*t*(45) = 9.06, *p* < 0.001, *d* = 2.70).

The main effects of the other two design variables were not significant (for valence *F*(2, 90) = 1.71, *p* = 0.19; *η*_*p*_^*2*^ = 0.04; for arousal *F*(2, 90) = 0.27, *p* = 0.77; *η*_*p*_^*2*^ = 0.006).

Moreover, we observed an interaction between valence and subjective significance (*F*(3.21, 144.46) = 2.65, *p* = 0.048, *η*_*p*_^*2*^ = 0.06). The overview of this interaction is presented in Figs. 2B and 2C. The *post hoc* tests showed that for neutral words, the pattern of differences between the levels of subjective significance followed the main effect. Namely, the amplitude was increasingly more positive with the significance level, and all the pairwise differences were significant. In the case of negative words, only the amplitude for low significant stimuli was less positive than for medium and highly significant words. The amplitude for the last two levels was not statistically different. The statistical details of this interaction are described in Appendix 2.

**Figure 3:**
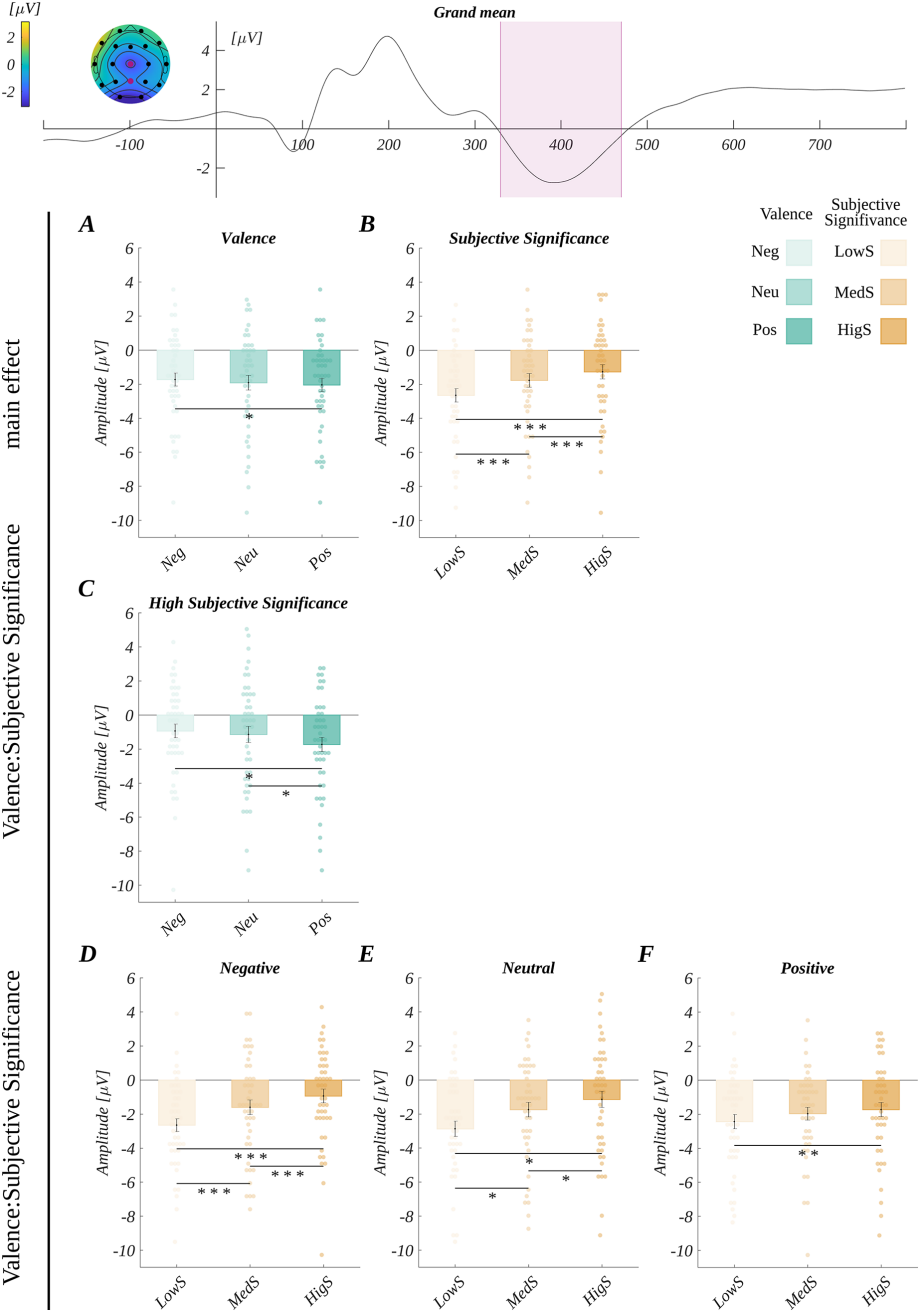
Grand average across electrodes from ROI_N400_ and results for N400 analysis. The upper plot: the grand average across electrodes from ROI_N400_. The marked time-interval was selected for the N400 analysis. The insert shows the topography of the mean potential from this period with the electrodes forming the ROI_N400_ marked with pink dots. Main effects for: (A) valence, (B) subjective significance. Interaction between valence and subjective significance: (C) differences between valence levels for highly significant words, and differences between subjective significance levels for: (D) negative, (E) neutral, and (F) positive words. Bars represent the mean value, error bars *SEM*, individual dots mark the average amplitude of an individual subject in the given condition, black horizontal lines indicate pairs of conditions with significantly different means (*p < 0.05, **p < 0.01, ***p < 0.001).

Furthermore, in ROI_N400_ we also observed a three-way interaction between valence, arousal and subjective significance (*F*(8, 360) = 3.29, *p* = 0.001; *η*_*p*_^*2*^ = 0.07). Visualisation of this effect and the statistical details are reported in Appendix 2. The grand mean from ROI_N400_ is shown in Fig. 3.

In the three-way ANOVA with repeated measures, we obtained the main effect of valence (*F*(2, 90) = 3.60, *p* = 0.031; *η*_*p*_^*2*^ = 0.07). Here, the amplitude for positive words (*M* = −2.05, *SEM* = 0.39) was significantly more negative than for the negative words (*M* = −1.73, *SEM* = 0.39; *t*(45) = 2.69, *p* = 0.030, *d* = 0.80) (Fig. 3A).

Furthermore, the main effect of subjective significance (*F*(2, 90) = 63.22, *p* < 0.001; *η*_*p*_^*2*^ = 0.58) was found. The amplitude became progressively less negative with the increasing level of subjective significance (Fig. 3B). That is, the amplitude for medium significant words (*M* = −1.77, *SEM* = 0.40) was significantly less negative than for low significant words (*M* = −2.65, *SEM* = 0.39 ; *t*(45) = 7.51, *p* < 0.001, *d* = 2.24). Next, the amplitude for highly significant words (*M* = −1.27, *SEM* = 0.42) was less negative than for medium significant words (*t*(45) = 3.93, *p* < 0.001, *d* = 1.17). Also, the amplitude for highly significant words was less negative than for low significant words (*t*(45) = 10.78, *p* < 0.001, *d* = 3.21).

The main effect for arousal (*F*(2, 90) = 0.08, *p* = 0.92; *η*_*p*_^*2*^ = 0.002) was not statistically significant.

Moreover, we observed an interaction between valence and subjective significance (*F*(4, 180) = 4.12, *p* = 0.003; *η*_*p*_^*2*^ = 0.08). The overview of this interaction is shown in Figs. 3C–3F.

The *post hoc* tests showed that in the group with high subjective significance the amplitude for positive words was more negative than for negative and neutral words. Further, for groups of different valence, there were differences in amplitude between the levels of subjective significance that followed the pattern of the main effect, *i.e*., the amplitude was progressively less negative with the increase of subjective significance, and all the pairwise differences were statistically significant. However, in the case of positive words, this pattern became weaker, *i.e*., only the difference between high and low significant words remained statistically significant. The statistical details of this interaction are reported in Appendix 2.

**Figure 4:**
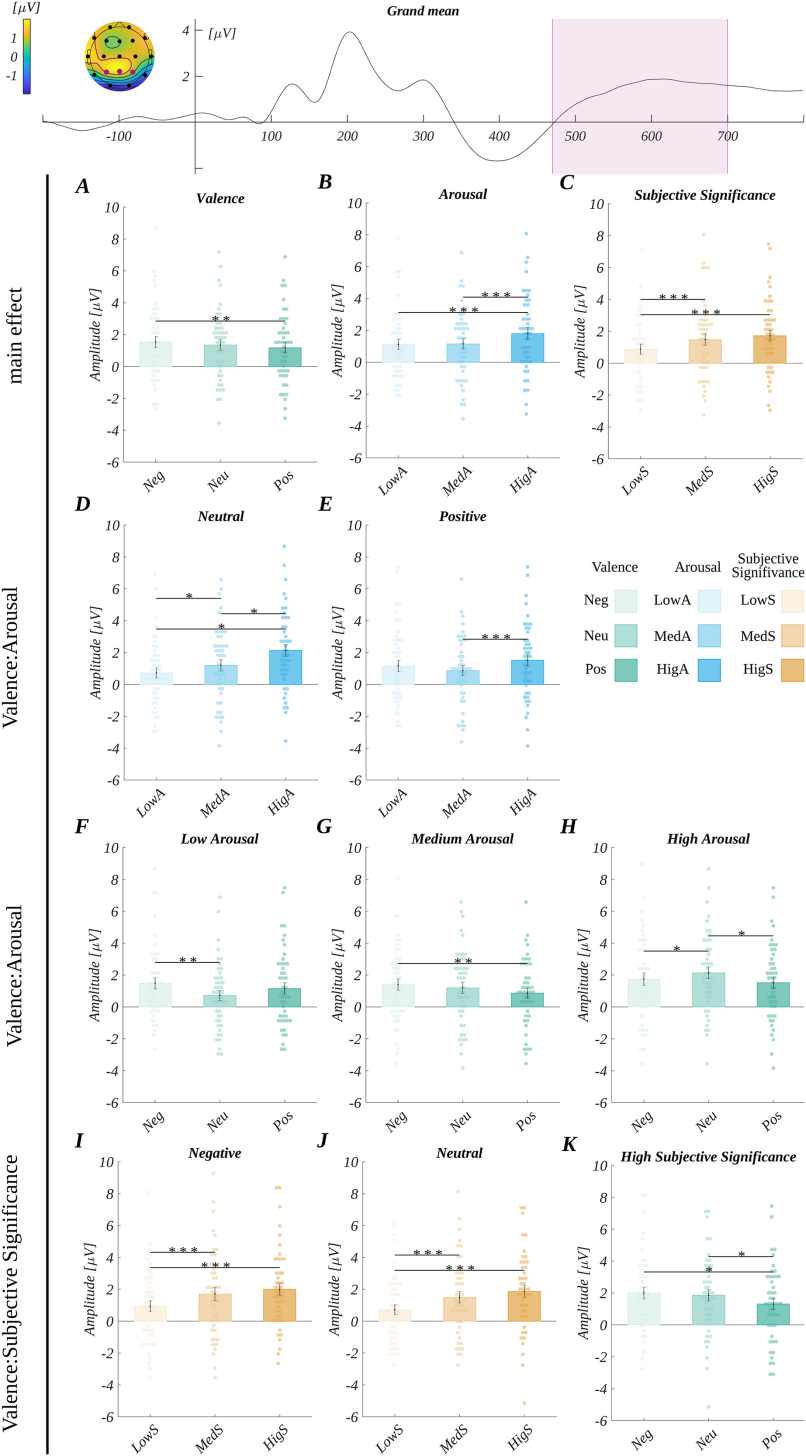
Grand average across electrodes from ROI_LPC_ and results for LPC analysis. The upper plot: the grand average across electrodes from ROI_LPC_. The marked time-interval was selected for the LPC analysis. The insert shows the topography of the mean potential from this period with the electrodes forming the ROI_LPC_ marked with pink dots. Main effects for (A) valence, (B) arousal, and (C) subjective significance. Interaction effects: differences between arousal levels for: (D) neutral and (E) positive words; differences between valence levels for: (F) low arousal, (G) medium arousal, and (H) high arousal condition; differences between subjective significance levels for: (I) negative and (J) neutral words; (K) differences between valence levels at high subjective significance level. Bars represent the mean value, error bars *SEM*, individual dots mark the average response time of an individual subject in the given condition, black horizontal lines indicate pairs of conditions with significantly different means (*p < 0.05, **p < 0.01, ***p < 0.001).

Additionally, in ROI_LPC_ we observed a three-way interaction between valence, arousal and subjective significance (*F*(8, 360) = 4.88, *p* < 0.001; *η*_*p*_^*2*^ = 0.10). The details of this analysis are reported in Appendix 2. The grand mean from ROI_LPC_ is shown in Fig. 4.

In the three-way ANOVA with repeated measures, the main effect of valence (*F*(2, 90) = 5.30, *p* = 0.007; *η*_*p*_^*2*^ = 0.11) was found. The amplitude for negative words (*M* = 1.53, *SEM* = 0.35) was more positive than for positive ones (*M* = 1.17, *SEM* = 0.33; *t*(45) = 3.40, *p* = 0.004, *d* = 1.01) (Fig. 4A).

Further, we obtained the main effect of arousal (*F*(2, 90) = 21.51, *p* < 0.001; *η*_*p*_^*2*^ = 0.32). Here, the amplitude for highly arousing words (*M* = 1.79, *SEM* = 0.36) was more positive than for both low arousing (*M* = 1.11, *SEM* = 0.32; *t*(45) = 5.33, *p* < 0.001, *d* = 1.59) and medium arousing words (*M* = 1.15, *SEM* = 0.33; *t*(45) = 6.28, *p* < 0.001, *d* = 1.87) (Fig. 4B).

Also, the main effect of subjective significance (*F*(2, 90) = 22.61, *p* < 0.001; *η*_*p*_^*2*^ = 0.33) was found. In this case, the amplitude for low significant words (*M* = 0.87, *SEM* = 0.32) was less positive than for both medium significant (*M* = 1.47, *SEM* = 0.36; *t*(45) = 4.94, *p* < 0.001, *d* = 1.47) and highly significant words (*M* = 1.71, *SEM* = 0.34; *t*(45) = 6.15, *p* < 0.001, *d* = 1.83) (Fig. 4C).

Moreover, we observed the effect of interaction between valence and arousal (*F*(3.19, 143.74) = 7.05, *p* < 0.001, *η*_*p*_^*2*^ = 0.14). The overview of this interaction is shown in Figs. 4D–4H. The *post hoc* tests showed that the amplitude was increasingly more positive with the rising level of arousal for neutral words and all pairwise differences were significant. However, in the case of positive stimuli, the only observed difference was a more positive amplitude for highly arousing than medium arousing words. More differences were visible when we analysed the amplitude at fixed arousal levels. At the low arousing stimuli level, the amplitude was more positive for negative than neutral words. In the case of medium arousing words, the amplitude for negative stimuli was more positive than for positive stimuli. Finally, the amplitude for neutral stimuli was more positive than for both negative and positive words for highly arousing stimuli. The statistical details of this interaction are reported in Appendix 2.

Furthermore, we obtained the effect of an interaction between valence and subjective significance (*F*(4, 180) = 3.40, *p* = 0.010; *η*_*p*_^*2*^ = 0.07). The overview of this interaction is shown in Figs. 4I–4K. The *post hoc* tests revealed that the pattern of amplitude differences between the subjective significance levels for negative and neutral words was the same as for the main effect, *i.e*., the amplitude for low significant stimuli was less positive than for both medium and highly significant words. However, we did not obtain any effect of subjective significance for words of positive valence. In the case of highly subjective significant words, the amplitude for positive stimuli was less positive than for both negative and neutral stimuli. The statistical details of this interaction are reported in Appendix 2.

Additionally, we observed the three-way interaction between valence, arousal and subjective significance (*F*(8, 360) = 6.85, *p* < 0.001; *η*_*p*_^*2*^ = 0.13). The details of this analysis are reported in Appendix 2.

#### Reaction times

**Figure 5:**
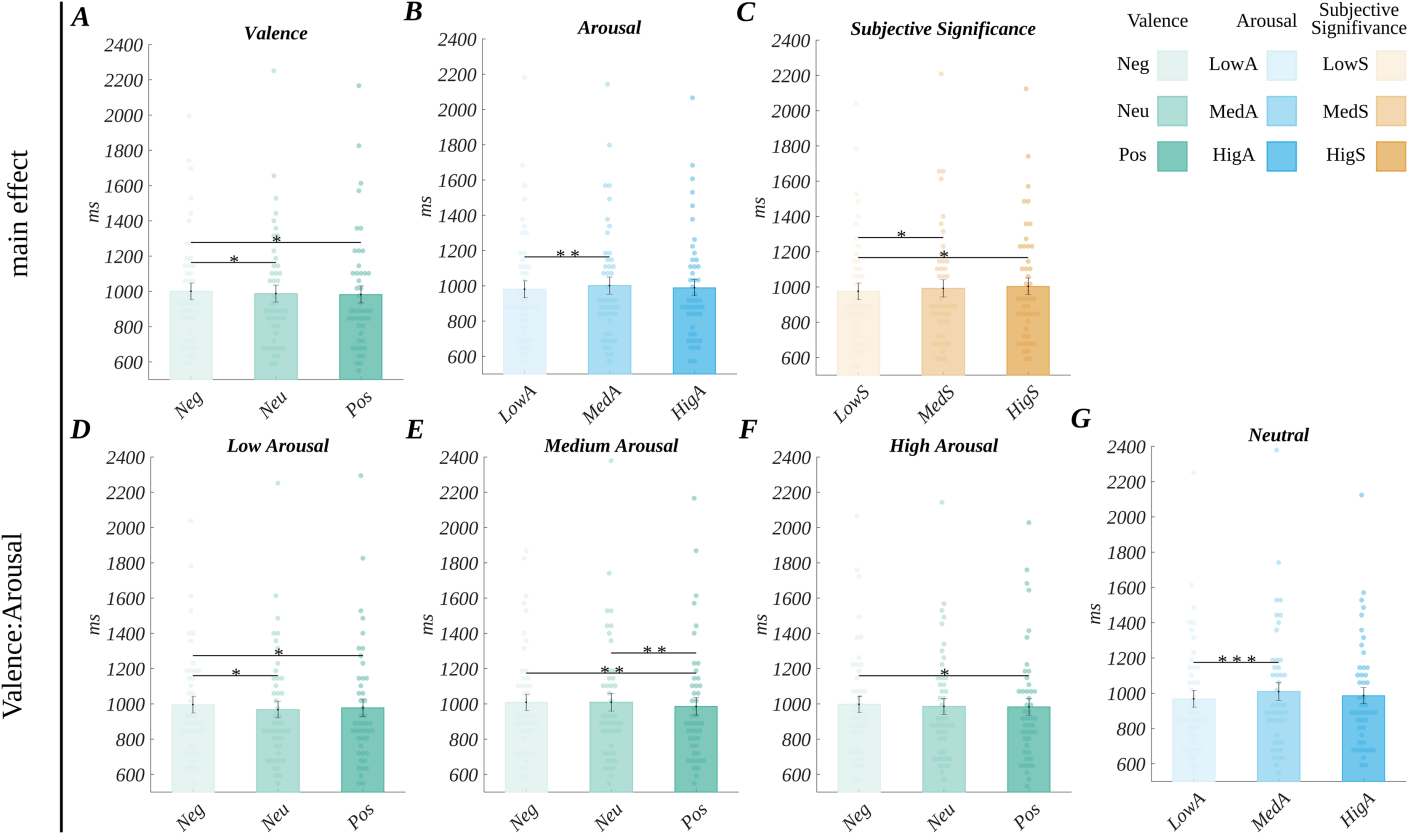
Main and interaction effects in reaction times. Top row main effects for: (A) valence, (B) arousal, (C) subjective significance; bottom row interaction effects, differences between valence levels at: (D) low arousal, (E) medium arousal, and (F) high arousal; (G) differences between arousal levels for neutral stimuli. Bars represent the mean value, error bars *SEM*, individual dots mark the average response time of an individual subject in the given condition, black horizontal lines indicate pairs of conditions with significantly different means (*p < 0.05, **p < 0.01, ***p < 0.001).

We investigated the reaction time (RT) dependence on the valence, arousal and subjective significance using three-way ANOVA with repeated measures. We used the natural logarithm transformation to make the distribution of response latencies closer to normal. We report the values of *M* and *SEM* in this section in milliseconds. We observed the main effects of all three factors. Namely, we found the main effect of valence (*F*(2, 90) = 10.29, *p* < 0.001; *η*_*p*_^*2*^ = 0.19). The *post hoc* tests showed that the reaction time was the longest for negative stimuli (*M* = 1,000.32, *SEM* = 46.25). It was longer than for neutral (*M* = 987.47, *SEM* = 47.86; *t*(45) = 2.80, *p* = 0.015, *d* = 0.840) and positive words (*M* = 981.84, *SEM* = 48.72; *t*(45) = 4.74, *p* < 0.001, *d* = 1.41) (Fig. 5A).

Moreover, we found the main effect of arousal (*F(*1.67, 75.12) = 4.91, *p* = 0.014, *η*_*p*_^*2*^ = 0.10). The *post hoc* tests (Fig. 5B) indicated that the participants’ reaction time for medium arousing words (*M* = 1,000.93, *SEM* = 48.50) was significantly longer than for the low arousing words (*M* = 979.95, *SEM* = 47.92; *t*(45) = 3.81, *p* = 0.001, *d* = 1.14).

We also observed the main effect of subjective significance (*F*(1.76, 79.33) = 8.34, *p* = 0.001, *η*_*p*_^*2*^ = 0.16). The *post hoc* tests (Fig. 5C) indicated that the reaction time for low significant words (*M* = 975.26, *SEM* = 46.49) was shorter than for highly significant words (*M* = 1,002.82, *SEM* = 47.30; *t*(45) = 3.57, *p* = 0.003, *d* = 1.06) and shorter than medium significant words (*M* = 991.55, *SEM* = 49.10; *t*(45) = 2.53, *p* = 0.030, *d* = 0.75).

Furthermore, we obtained an interaction between valence and arousal (*F*(4, 180) = 2.98, *p* = 0.021; *η*_*p*_^*2*^ = 0.06). For low arousing stimuli, the pattern of differences in RT between valence levels mimics the one observed for the main effect of valence. For medium arousing stimuli, the RT for neutral words was longer, and the difference in respect to negative stimuli became non-significant; however, the difference between the RT for negative and positive words became significant. In the case of highly arousing words, only the difference in RT between negative and positive stimuli was significant. Additionally, in the case of the neutrally valenced words, we observed longer RTs for medium *vs* low arousing stimuli, which mimics the pattern obtained for the main effect of arousal. The visualisation of the valence: arousal interaction is shown in Figs. 5D–5G; details of statistical tests are presented in Appendix 2. We also obtained a three-way interaction concerning the reaction times, as detailed in Appendix 2.

#### Frequency of emotional decisions

We analysed the influence of the manipulated factors on the perception of stimuli as emotional by the participants, measured as the frequency at which stimuli in a given experimental condition were classified as emotional, which will be further called the frequency of emotional decisions (FED). We used three-way ANOVA with repeated measures. We obtained significant main effects of all three factors.

**Figure 6:**
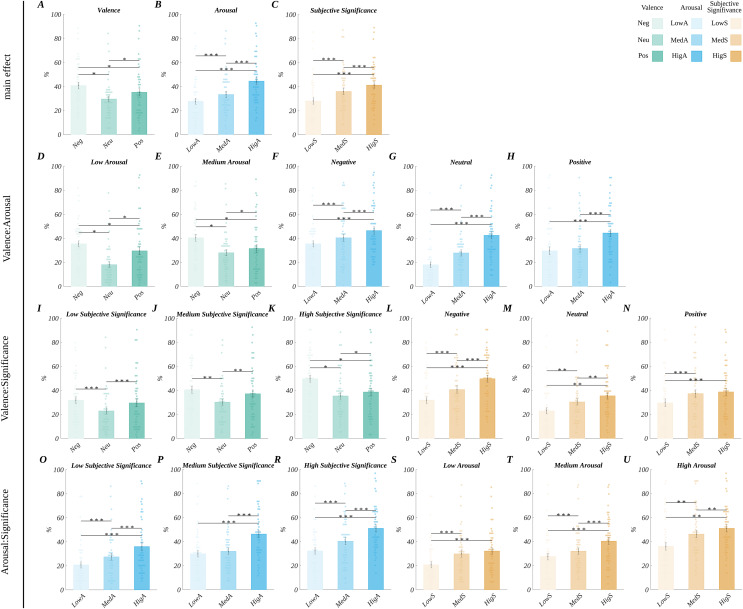
The differences in frequency of emotional decision. According to the levels of: (A) valence, (B) arousal, and (C) subjective significance. Second row, interaction effects: differences between valence levels at: (D) low arousal and (E) medium arousal; differences between arousal levels at (F) negative, (G) neutral, and (H) positive words. Third row, interaction effects: differences between valence levels at: (I) low subjective significance, (J) medium subjective significance, and (K) high subjective significance; differences between arousal levels at (L) negative, (M) neutral, and (N) positive words. Fourth row, interaction effects: differences between arousal levels at: (O) low subjective significance, (P) medium subjective significance, and (R) high subjective significance; differences between subjective significance levels at: (S) low arousal, (T) medium arousal, and (U) high arousal. Bars represent the mean value, error bars *SEM*, individual dots mark the average response time of an individual subject in the given condition, black horizontal lines indicate pairs of conditions with significantly different means (*p < 0.05, **p < 0.01, ***p < 0.001).

We obtained the main effect of valence (*F*(1.32, 59.50) = 22.67, *p* < 0.001, *η*_*p*_^*2*^ = 0.33). Here, the *post hoc* test indicated (Fig. 6A) that participants tended to classify neutral words as emotional less frequently (*M* = 29.50, *SEM* = 2.47) than both negative (*M* = 40.66, *SEM* = 2.75; *t*(45) = 8.35, *p* < 0.001, *d* = 2.49) and positive words (*M* = 35.09, *SEM* = 3.07; *t* (45) = 4.23, *p* < 0.001, *d* = 1.26). Also, positive stimuli were classified as emotional less frequently than the negative ones (*t*(45) = −2.56, *p* = 0.014, *d* = 0.76).

Furthermore, we obtained the main effect of arousal (*F*(1.21, 54.42) = 91.61, *p* < 0.001, *η*_*p*_^*2*^ = 0.67). The *post hoc* tests showed (Fig. 6B) that the participants tended to classify the stimuli as emotional more frequently, the more arousing the stimuli were. Namely, FED was significantly lower for low arousing (*M* = 27.59, *SEM* = 2.55) than for the medium (*M* = 33.24, *SEM* = 2.64; *t*(45) = 6.93, *p* < 0.001, *d* = 2.07) and high arousing words (*M* = 44.42, *SEM* = 2.92; *t*(45) = 9.98, *p* < 0.001, *d* = 2.98). Also, FED was lower for medium arousing, than for high arousing words (*t*(45) = 9.82, *p* < 0.001, *d* = 2.93).

Finally, we obtained the main effect of subjective significance (*F*(1.22, 54.68) = 47.92, *p* < 0.001, *η*_*p*_^*2*^ = 0.52). The *post hoc* tests showed (Fig. 6C) that the participants more frequently classified words with higher subjective significance as emotional. FED was lower for low significant words (*M* = 28.05, *SEM* = 2.73) than for medium significant (*M* = 36.03, *SEM* = 2.70; *t*(45) = 10.04, *p* < 0.001, *d* = 2.99) and highly significant ones (*M* = 41.17, *SEM* = 2.73; *t*(45) = 7.38, *p* < 0.001, *d* = 2.20). Also, FED was lower for medium than for highly significant words (*t*(45) = −3.97, *p* < 0.001, *d* = 1.18).

Moreover, we observed an interaction between valence and arousal (*F* (3.02, 136.01) = 20.55, *p* < 0.001, *η*_*p*_^*2*^ = 0.31). The overview of this interaction is shown in Figs. 6D–6H. The *post hoc* tests showed that the pattern of differences between valence levels, in the case of low and medium arousing words, mimicked the pattern observed for the main effect of valence. Namely, FED was the highest for negative words and the lowest for neutral ones, and the differences between all valence levels were significant. We did not observe significant differences between the valence levels in the case of highly arousing stimuli.

When we considered the differences between arousal levels at fixed valence levels, we found that FED increased with increasing arousal of the stimuli for negative and neutral valenced words, with all valence levels being significantly different, *i.e*., reflecting the main effect of arousal. For positive stimuli, we observed that FED was significantly higher only for high arousal rather than for low and medium arousal words. The statistical details of this interaction are reported in Appendix 2.

Furthermore, we observed an interaction between valence and subjective significance (*F* (4, 180) = 8.24, *p* < 0.001; *η*_*p*_^*2*^ = 0.15). The overview of this interaction is shown in Figs. 6I–6N. The *post hoc* tests showed that for the high level of subjective significance, the pattern of differences between the valence levels was the same as for the main effect of valence. Namely, FED was the highest for negative, moderate for positive, and the lowest for neutral stimuli and all the pairwise differences were significant. On the other hand, in the case of stimuli with low and medium subjective significance, FED for negative and positive words equalized (the difference was not significant), and it was significantly higher than for neutral stimuli. Comparing the subjective significance levels at fixed valence levels, we observed that FED increased with the level of subjective significance for negative and neutral words, and all the pairwise differences were statistically significant. It was analogous to the main subjective significance effect. In the case of positive stimuli, the FED for medium significant words equalized with highly significant ones, and the difference between these levels was not statistically significant. The statistical details of this interaction are reported in Appendix 2.

Lastly, we observed an interaction between arousal and subjective significance (*F*(4, 180) = 5.58, *p* < 0.001; *η*_*p*_^*2*^ = 0.11). The overview of this interaction is shown in Figs. 6O–6U. The *post hoc* tests for fixed levels of subjective significance revealed that the participants classified more arousing words as emotional more frequently, in the case of low and high subjectively significant stimuli. Each pairwise difference was statistically significant; this was the pattern observed for the main effect of arousal. In the case of medium subjective significance, FED for highly arousing words was higher than for low and medium arousing ones, but it equalized between low and medium arousing stimuli.

When we fixed arousal levels, the *post hoc* tests showed that in the case of medium and highly arousing words, participants classified them as emotional more often if they were of higher subjective significance. Each pairwise difference was significant, mimicking the main effect of subjective significance. In the case of low arousal, FED for medium and high subjective significance equalized and was higher than for low subjective significant words. The statistical details of this interaction are reported in Appendix 2.

## Discussion

In the current experiment, we expected to find an impact of subjective significance, valence and arousal of emotion-laden words on explicit emotionality processing in the emotional categorisation task. We found emotional factors to influence three subsequent ERP components (P300, N400 and LPC), as well as reaction times and the results of the task.

The amplitude for the P300 component was significantly increased along with the increasing subjective significance. This result seems to be in line with some previous findings, presenting how the meaningfulness of the stimuli may shape the amplitudes of the components (Johnson, 2007; Thomas, Johnstone & Gonsalvez, 2007). We also found an interaction effect between the valence and subjective significance; for negative words, significant differences were present amongst all significance levels (with amplitude being the most positive for highly significant stimuli). For neutral words, differences were between weakly significant stimuli and both moderately and highly significant ones; and for positive stimuli, there was only a difference between low and high significance. That is particularly interesting, as such differences in the processing of word stimuli may show how the valence and subjective significance can shape the affective experience (van Hooff et al., 2008). For negative stimuli, even a small change in subjective significance is crucial and elicits a more attenuated response, whereas for positive valence, recognising high and low significance seems to be enough–this however may be an effect specific to the set of stimuli used in our study, and eventual generalization of this effect could only be done after replication in further studies. In the three-way interaction we mainly observed the amplification of the interaction between subjective significance and valence for low and medium arousing stimuli with different levels of subjective significance shaping the differences between amplitudes for negative words, however we also observed high arousal shaping the effects of subjective significance among the positive words (see Appendix 2).

For the N400 component, we found the main effect of valence; namely, the amplitudes for positively valenced stimuli were significantly more negative than for negative words, which is in contradiction with most of the reported results regarding the influence of emotional valence on this component (Federmeier et al., 2001; Kanske & Kotz, 2007; De Pascalis et al., 2009; Yao et al., 2016). This could be explained by the lack of extremely negative words in the group of experimental stimuli, which was caused by manipulating three factors simultaneously, which is further discussed in the Limitations. We also obtained a main effect of subjective significance, with negativity of wave decreasing gradually with the level of subjective significance. This effect is congruent with our previous results (Imbir et al., 2018b). N400 may be interpreted as the component indicating the perception of surprise caused by unexpected stimuli (Reid et al., 2009; Kutas & Federmeier, 2011; Bridger et al., 2012), non-significant words may be unexpected in the context of the set of stimuli consisting of emotional words, thus evoking more negative amplitudes than the significant ones. More significant words may also be easier to process for participants, as they are more relevant for participants and most likely have been previously processed in the emotional context. This effect may also have another explanation, related to the specificity of the subjective significance of stimuli. This factor is strongly correlated with the concreteness dimension, with more significant words also being assessed as more abstract (Imbir, 2016c), and concrete words have been shown to elicit more negative N400 amplitudes than abstract ones (Kanske & Kotz, 2007), similarly to words with low significance in our study. The differences between particular word groups within the interaction effects were mostly a reflection of main effects; the emotional charge brought by the highly significant positive words may, however, explain the observed main effect of valence.

As LPC is the component most associated with an explicit processing of emotionality (Sass et al., 2010; González-Villar et al., 2014), it is here that the observed emotional modulation should most resemble the judgements of emotionality. Accordingly, the shapes of the main effects of subjective significance and arousal both resembled their respective patterns of emotional decisions—the amplitudes were gradually more positive with increasing levels of these activation factors. This again may confirm the similarity of influence these factors have on processing, as it is also supported by shapes of effects for neutral words being similar to main effects. These replicated shapes of differences have been observed in two-way interactions of both factors with valence, as well as in three-way interactions for neutral words, for subjective significance for all levels of arousal and for arousal for all levels of subjective significance (see Appendix 2). It is important to note, that at this late stage of processing we can see the main effect of arousal, which was not present earlier. This seems to be in line with previous results reporting valence to influence processing earlier than arousal (Olofsson et al., 2008). As for valence, we observed no differentiation between neutral and valenced conditions—a divergence from emotionality judgements. Valence produced a more positive amplitude for negative words, when compared to positive words.

Besides the discussed effects of subjective significance, controlling this factor allowed us to observe the modulating interaction effect of arousal on the shape of the valence effect. For words with high arousal, neutral words produced a more positive amplitude than both negative and positive words. For moderate arousal, valence produced a different pattern that resembled the main effect, with a more positive amplitude for negative when compared to positive words. However, for words with low arousal, negative words produced a more positive amplitude than neutral words. This means that the amplitude of neutral words was more and more negative as we walked down the levels of arousal. Note how averaging these effects may explain the lack of the main effect of valence, seen in similar studies (González-Villar et al., 2014; Delaney-Busch, Wilkie & Kuperberg, 2016), in particular in those that did not examine this interaction.

To sum up the ERP results—subjective significance effects were observed throughout the whole span of the analysed waveform, while other emotional factors played a role in separate stages of making the decision about emotionality. High subjective significance evoked more positive amplitude in the P300 component, mainly related to the decision-making, and the wave stayed less negative through the N400 component, where more significant words could have been less surprising for participants. In the LPC component, high subjective significance still evoked more positive amplitudes, which is related to the greater emotional load of significant words, as in this late component the emotional value of words could be reflected on by participants. Positive valence reduced the effects of subjective significance in the P300, positively valenced words also evoked more negative amplitudes in the N400 component. However, in the LPC component it was the negative valence that caused more positive deflection, which is in line with longer reaction times caused by negative valence—the negative emotions caused greater disruption in late stages of processing. The same could be said about emotional arousal, the influence of which was observed only in the LPC component, with highly arousing words evoking more positive amplitudes than less arousing ones. As for the complex interactions of all three factors, we were mainly interested in valence and arousal modulating the effects of subjective significance. Valence magnified the effects of subjective significance among low and medium arousing words, which could be especially seen among the negative words, we have also observed that varying levels of subjective significance were modulated by arousal mostly for non-neutral words.

Considering the behavioural results, the words high in subjective significance evoked longer reactions than those low on this factor, which is congruent with our previous findings (Imbir et al., 2020a). We also found differences regarding valence, namely the reaction times after negative words were longer than in both neutral and positive conditions. This result is also congruent with previous findings (Hinojosa, Méndez-Bértolo & Pozo, 2010; Imbir et al., 2020a, 2021b). What is more, the effect of valence was observed in all levels of arousal separately, which indicates the considerable influence of negative emotions on the speed of cognitive processing. Regarding arousal, we found that the words with a low level of arousal evoked the shortest reaction times, which is also congruent with previous findings (Yao et al., 2016; Imbir et al., 2020a). The words low on the arousal scale are close to being non-emotional, thus not initiating the affective charge, which would slow down the processing of the decision.

For the frequencies of emotional decisions we identified two similar effects regarding arousal and subjective significance: the higher the level of variable, the more frequently was the word marked as emotional. Both of those effects reflected the previously described differences observed in the LPC component. When it comes to subjective significance, this could also explain the effect on reaction times, as the greater emotional charge was causing a slowdown in reactions. This result could also indirectly support the proposed similarity between the two dimensions for different levels of processing—automatic and reflective (Imbir, 2016a)—which is further supported by the shapes of results being replicated for negative, neutral and positive words separately (see Appendix 2). The behavioural results regarding the frequency of emotional decision have also proven the validity of the stimuli selection and were congruent with our hypotheses. The emotionally valenced word groups were ranked as more emotional than those marked as neutral. Moreover, negative words were ranked as more emotional than positive ones, which is congruent with previous studies (Schrauf & Sanchez, 2004). This effect could be explained by the negative words imposing greater impact than the positive ones, as the negative words are related to aversive concepts, which should focus attention in order to be avoided. This is also congruent with the results regarding reaction times, where the negative words lengthened the times of making the decision. The main observation from the three-way interaction may be, that high levels of all three emotional factors increased the number of words marked as emotional.

Obtained results support the hypothesis about the possibility to control all three emotional factors in the orthogonal study design, which might be relevant for future studies. The effects regarding high subjective significance seem especially important (slowing down reaction times, evoking more positive/less negative amplitudes throughout all components), as the factor was only recently proposed as an important dimension for emotional processing (Imbir, 2016a). The multidimensional approach also allowed us to identify a number of interactions between emotional factors, with interactions of all three manipulated factors. Aside from layered interactions, the role of subjective significance itself in decision-making needs further exploration, as this is one of the first studies to verify the influence of this factor in a task requiring emotional decisions (Imbir et al., 2018b).

### Limitations

The first limitation of this study was the age of the participants, being mostly young adults, which limits the generalisation of the results. On the other hand, it enabled the sample to be coherent, as we avoided differences in neural processing stemming from development in later adulthood. Additionally, the invited sample age was coherent with the age of participants assessing the selected verbal stimuli (Imbir, 2016c), and thus allowed us to generalise the understanding of valence, arousal, and especially subjective significance in a generational way. Another limitation is the number of dimensions used in the orthogonal manipulation. We put much effort into preparing orthogonal manipulations, even though in the real world, emotional dimensions are correlated, often in a nonlinear manner, *i.e*., valence and arousal (Russell, 1980; Lang, Bradley & Cuthbert, 1997; Imbir, 2016c). The orthogonal manipulations are crucial for unambiguous interpretation of the results. However, we have to keep in mind, that due to using this way of words selection, the stimuli used in the study were rather unpolarised in the intensity of affective ratings. It also has to be mentioned that the design in which three factors are simultaneously divided into three levels is close to the threshold of a rational interpretation of the results. Following particular interactions within a three-factorial space could be rather labyrinthine, which is one of the reasons why results concerning such interactions have not been presented in the main body of this article. However, reducing the number of variables (or levels into which the variables are divided into) would reduce both the novelty and theoretical validity of the study. The applied approach allowed for a unique, comprehensive investigation of explicit processing of emotionality.

We also selected the stimuli to match in length and frequency of use, as these properties may significantly influence the perception of words (Graf, Nagler & Jacobs, 2005). Selecting stimuli in this manner creates a specific sample, which does not reflect the correlations between semantic properties of words, their length and frequency of use. A more ecologically accurate approach to analyse emotional factors of words processing with other factors controlled may be megastudies and virtual experiments, which allow the analysis of a much larger sample of words (*e.g*., Kuperman et al., 2014; Kuperman, 2015). Other approaches to studying linguistic material may also allow the control of more semantic properties of words at once, such as concreteness or imageability, which also have been shown to influence the processing of words (Kanske & Kotz, 2007; Siakaluk, Knol & Pexman, 2014).

A more ecologically accurate approach to the study design should also include a sample of linguistic stimuli, whose emotional properties reflect the correlations present in the natural language. As was mentioned before, emotional valence and arousal are correlated (Russell, 1980; Lang, Bradley & Cuthbert, 1997; Bradley & Lang, 1999; Imbir, 2016c), while in the present study they were crossed orthogonally to create separate groups of high arousal and neutral valence or low arousal and positive valence, which are either very small or close to non-existent in natural language. The actual structure of language should be considered when designing future experiments exploring the influence of emotional words. These limitations in stimuli selection also led to a rather small sample of stimuli (15 words) for each group, thus the groups of words had to be presented twice to obtain reliable ERP data (Luck, 2014). The reaction times and ERP amplitudes were averaged between the first and the second presentation of the stimuli, which does not allow to observe the differences between the two series. We did not observe significant changes between frequency of emotional ratings between the two blocks, however changes in behavioural and neurophysiological response to the stimuli between the first and second presentation might be interesting to explore in future studies, especially in relation to subjective significance, which, as mentioned earlier, is strongly related to the semantic familiarity of the stimuli.

The neurophysiological measures used in this study could also be improved in future experiments. More early components should be analysed, such as early posterior negativity (EPN), in order to investigate the role of emotional factors in early stages of processing words (Frühholz, Jellinghaus & Herrmann, 2011). As for the current study, two consecutive components—P300 and N400—have been analysed using the same set of electrodes located in the centro-parietal area of the scalp. This may result in interpreting processes from these two components as two parts of one, longer process. On the contrary, we observed different effects in both of these components, with valence influencing only the latter component. Future studies should put more focus on the exact, theory-driven selection of electrodes for each component, in order to avoid confusion and provide the possibility for unambiguous interpretation of the results.

## Conclusions

We wanted to present some practical implications of the results shown in this article. The effects of subjective significance observed throughout the whole span of processing, as well as the effects on the behavioural level, suggest the importance of this factor for people while analysing objects in the surrounding world. This conclusion may be easily incorporated to the practical field—situations such as therapy, cognitive training or coaching. The fact that significant stimuli are processed differently to non-significant ones may explain some issues appearing during therapy or coaching. Consequently, it may also lead to better preparation of therapeutic programmes, with focus on topics significant for clients (or the proper proportion between significant and non-significant topics) and the possibility to better understand and manage clients’ potential reactions to those topics.

As mentioned before, it was proposed that subjective significance could be interpreted as the activation factor, similarly to arousal. This means that while a moderate charge of subjective significance could facilitate processing (which probably could be seen in the results that we present, as it is not easy to reach high subjective significance with linguistic stimuli not related to particular participants), the high charge should disrupt cognitive processes. When incorporating conclusions from our studies into the practical field it should also not be forgotten that the subjective significance interacts with emotional valence, similarly to arousal, which also may change the interpretation of different stimuli or topics by clients in, for example, the therapeutic process.

Besides the subjective significance, we have also found the main effects of valence and arousal influencing the decisions, reaction times and ERPs at different stages of processing, which confirms our hypotheses and is mostly in line with previous findings (Schrauf & Sanchez, 2004; Hinojosa, Méndez-Bértolo & Pozo, 2010; González-Villar et al., 2014; Delaney-Busch, Wilkie & Kuperberg, 2016). We observed the timeline of different emotional variables influencing processing, with subjective significance playing its role at the beginning, followed by valence and arousal, however this timeline has to be interpreted keeping in mind that all the three factors have been interacting with each other from the first analysed component (P300). The interactions, due to their complexity, may be difficult to interpret, however the main takeaway message from them may be that subjective significance and arousal create similar shapes of results, especially for neutral stimuli. The study has its limitations, the description of which can work as a set of guidelines for future research exploring decisions regarding emotionality of stimuli. The results show that subjective significance is an essential factor for understanding the structure of affect and affect consequences for cognition.

## Supplemental Information

10.7717/peerj.14583/supp-1 Supplemental Information 1 Word stimuli used in the experiment. The list of all words used in the experiment with their affective properties and level of each manipulated variable assigned to them; ANOVA analyses of differences between word groups on different affective scales; ANOVA analyses of affective properties - within differently valenced groups; groups of different arousal; subjective significance; Descriptive statistics of all word groups on manipulated (affective) and controlled scales.

10.7717/peerj.14583/supp-2 Supplemental Information 2 Results - exact statistics for main effects and interactions. The exact statistics (descriptive and inferential) for main effects, two- and three-way interactions for the P300 component; N400 component; LPC component; reaction times; frequency of emotional decision.

## Additional Information and Declarations

### Data Availability

The following information was supplied regarding data availability: The datasets generated during and/or analysed during the current study are available at Figshare: Imbir, Kamil (2022): Dataset: Arousal, subjective significance and the origin of neutrally valenced words in the processing of an emotional categorisation task (E6). figshare. Dataset. https://doi.org/10.6084/m9.figshare.14680506.v1.
